# Alteration of Diffusion Capacity After SARS-CoV-2 Infection: A Pathophysiological Approach

**DOI:** 10.3389/fphys.2021.624062

**Published:** 2021-03-25

**Authors:** Justine Frija-Masson, Catherine Bancal, Laurent Plantier, Hélène Benzaquen, Laurence Mangin, Dominique Penaud, Florence Arnoult, Martin Flamant, Marie-Pia d’Ortho

**Affiliations:** ^1^Neurodiderot, INSERM, Université de Paris, Paris, France; ^2^Physiologie-Explorations Fonctionnelles, FHU APOLLO, Assistance Publique Hôpitaux de Paris, Hôpital Bichat-Claude Bernard, Paris, France; ^3^Centre de Ressources et de Compétences de la Mucoviscidose, Service de Pneumologie et Explorations Fonctionnelles Respiratoires, CHRU de Tours, Tours, France; ^4^CEPR/INSERM, UMR 1100, Université de Tours, Tours, France; ^5^Laboratoire Matière et Systèmes Complexes, UMR 7505, CNRS, Paris, France; ^6^INSERM U 1149, Center for Research in Inflammation, Université de Paris, Paris, France

**Keywords:** SARS-CoV-2, *D*_LCO_, pneumonia, pulmonary function test, COVID-19

## Abstract

Severe acute respiratory syndrome coronavirus 2 (*SARS*-*CoV*-*2*) infection has affected millions of people worldwide, and pneumonia affects 90% of patients. This raises the possibility of millions of people with altered lung function. Few data exist to date on pulmonary function after SARS-CoV-2 infection, but alteration of diffusion capacity of CO (*D*_LCO_) is the most frequently described abnormality. First, we present original data on lung function at 3 months after SARS-CoV-2 infection and discuss the effect of using European Coal and Steel Community (ECSC) or Global Lung Function Initiative (GLI) reference equations to diagnose diffusion capacity. Second, we review existing data on *D*_LCO_ alteration after SARS-CoV-2 infection and discuss the implication of restrictive disorder in *D*_LCO_ alteration. Last, we discuss the pathophysiology of *D*_LCO_ alteration and try to disentangle vascular damage and fibrosis.

## Introduction

Severe acute respiratory syndrome coronavirus 2 (SARS-CoV-2) infection has affected more than 100 million of people worldwide and more than 60 million have recovered (Jonhs Hopkins University of Medicine, 2018). Pneumonia affects more than 90% of patients and can range clinically from asymptomatic to acute respiratory distress syndrome. The radiological extent of pneumonia can be classified as absent, mild (<10% of parenchyma involved), moderate (10–24%), wide (25–49%), severe (50–74%), or very severe (>75%), according to European guidelines (Revel et al., 2020). The high number of affected people raises concern about the possibility of having millions of people with altered pulmonary function tests (PFTs). To date, few data exist on the frequency of clinically relevant PFT abnormalities after coronavirus disease 2019 (COVID-19), but alteration of diffusion capacity (*D*_LCO_) is the most frequently described feature (Frija-Masson et al., 2020; Mo et al., 2020; Zhao et al., 2020a). However, PFT are not accessible worldwide, and a better knowledge of the pathophysiology underlying *D*_LCO_ alteration could help prioritize patients for PFT access. In this article, we review existing data on PFT results after SARS-CoV-2 infection, discuss the possible pathophysiology of *D*_LCO_ alteration, and present original data on the importance of using appropriate reference equations for assessing normality of PFT.

## Diffusion Capacity Alteration After COVID-19 Is Frequent and Independent of Initial Clinical Severity

The transfer (or diffusion) conductance of the lung for carbon monoxide (*D*_LCO_) is measured using the single apnea method, by multiplying two values that can be considered independent, namely (1) the alveolar volume (VA), which is the lung volume where inhaled helium diffuses in gas state following inspiration from residual volume to total lung capacity and (2) the carbon monoxide transfer coefficient (kCO), which is the rate constant for carbon monoxide uptake in the lung. Thus, *D*_LCO_ can be reduced by reduction in lung volumes (restriction) or reduction in carbon monoxide uptake. Since carbon monoxide uptake depends on the integrity of the alveolar–capillary membrane and the presence of hemoglobin across the alveolar–capillary membrane, any alteration in either alveolar lung regions or the pulmonary vasculature results in reduced *D*_LCO_. In addition, although kCO increases with reductions in lung inflation, this relationship is not proportional (Johnson, 2000); thus, restriction due to reduced chest wall compliance or respiratory muscle weakness may result in reduced *D*_LCO_. Since *D*_LCO_ depends on the integrity of almost all structures of the respiratory system (conducting airways excluded), it is highly sensitive to detect lung disease. Because kCO depends on lung inflation, it is difficult to interpret in restrictive lung disease where low lung volumes may result from reductions in chest wall compliance, inspiratory muscle weakness, or reduced lung compliance.

### The Importance of Choosing the Right Predicted Values

We conducted a retrospective study on patients with confirmed SARS-CoV-2 infection (PCR) referred at 3 months after symptom onset for pulmonary PFT. Patients were referred to the PFT laboratory if they had presented with severe COVID-19 (i.e., had required at least 6 L/min oxygen or mechanical ventilation during acute infection) or if they still had respiratory symptoms at 3 months. Comparisons between groups used Mann–Whitney and Kruskal–Wallis (with Dunns’ multiple comparisons tests) tests for continuous variables and chi-2 or Fisher’s exact tests for categorical variables (Prims 8, Graphpad, San Diego, United States). Non-opposition was obtained for all patients, according to French law. The study was approved by the Institutional Review Board of the French Learned Society for Respiratory Medicine—Société de Pneumologie de Langue Française (ref 2020-056).

We included 146 patients [median age, 58 (Q1 = 49; Q3 = 67); median body mass index (BMI), 27.15 kg/m (24.72;30.35), 89 (61%) men]. Complete characteristics of patients are presented in Table 1.

**TABLE 1:** Patients’ characteristics.

	**All (*n* = 151)**
Age, years	57(49;67)
Male sex	91 (60)
BMI, kg/m^2^	27.29(24.72;30.35)
**Respiratory comorbidities**	
Asthma	10 (7)
COPD	9 (6)
Bronchiectasis	2 (1)
Sarcoidosis	3 (2)
Idiopathic pulmonary fibrosis	1 (0.01)
Lymphangioleiomyomatosis	1 (0.01)
Lung transplant	1 (0.01)
**Hypertension**	25 (17)
**Smoking status**	
Active	11 (7)
Former	9 (6)
**Respiratory support**	
None	37 (25)
Oxygen 0–6 L/min	61 (40)
Oxygen > 6 L/min	13 (8)
High flow nasal canula/CPAP	14 (9)
Invasive ventilation	26 (17)
**Initial pneumonia on chest CT**	
No chest CT	22 (15)
Absent	3 (2)
Mild	13 (8)
Moderate	37 (25)
Wide	42 (28)
Severe/extremely severe	34 (23)
**Pulmonary function tests**	
FEV_1_ (% pred)	109(91;119)
FVC (% pred)	109(93;119)
FEV_1_/FVC	0.72(0.71;0.73)
TLC (% pred)	103(90;115)
*D*_LCO_ (CECA,% pred)	71(69;72)
*D*_LCO_ (GLI,% pred)	74(63;84)

*Results are presented as median (Q1;Q3) for continuous variables or n (%) for categorical variables. Extent of pneumonia on initial CT was defined as absent, mild (<10%), moderate (10–24%), wide (25–49%), or severe/extremely severe (≥50%) (Revel et al., 2020).*

**FIGURE 1:**
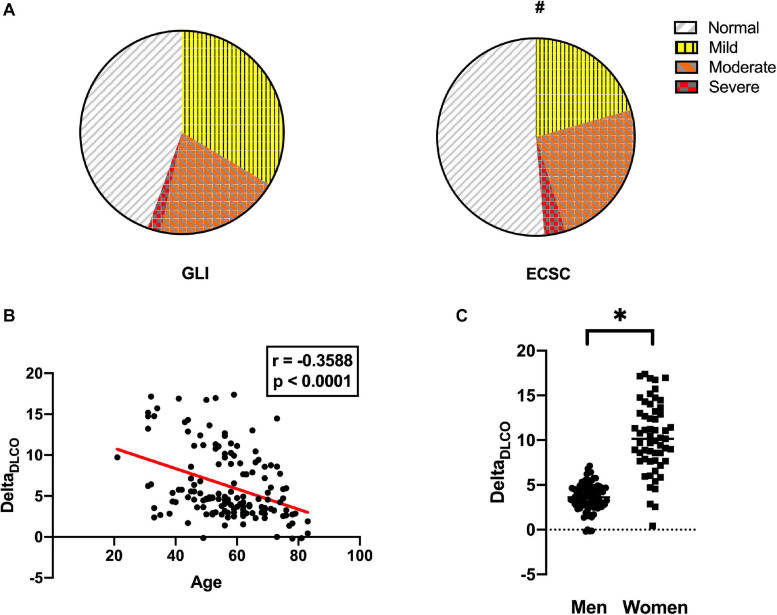
Comparison between European Coal and Steel Community (ECSC) and Global Lung Function Initiative (GLI) reference equation in the diagnosis of diffusion capacity alteration. **(A)** Proportion of patients with normal *D*_LCO_, mild, moderate, or severe diffusion alteration according to GLI (left) or ECSC (right) reference equations; ^#^*p* < 0.05 (Mann–Whitney). **(B)** Spearman correlation showing the effect of age on Delta_DLCO_ (*D*_LCO_%_pred GLI_ – *D*_LCO_%_prced ECSC_). **(C)** Effect of sex on Delta_DLCO_; ^∗^*p* < 0.0001.

To assess the effect of reference equations on altered diffusion capacity prevalence, we determined percent of predicted value for European Coal and Steel Community (ECSC) 93 (Pellegrino, 2005) and Global Lung Function Initiative (GLI) (Stanojevic et al., 2017) reference equations; altered diffusion capacity was defined as *D*_LCO_ < LLN. Median (% pred) *D*_LCO_ were 70 (69;72) for ECSC 93 and 72 (63;84) for GLI, *p* < 0.0001. Interestingly, when using ECSC 93 reference equations, 71 (49%) patients had altered diffusion capacity (among which light *n* = 30, moderate *n* = 36, severe *n* = 5), while 76 (54%) patients were diagnosed with altered *D*_LCO_ when using GLI reference equations (light *n* = 49, moderate *n* = 29, severe *n* = 3, *p* = 0.0231 for disease severity) (see Figure 1).

We determined the difference in predicted value between GLI and ECSC 93: Delta_DLCO_ = *D*_LCO__%__pred GLI_ − *D*_LCO_%_pred ECSC_. There was an inverse correlation between age and Delta_DLCO_ with a Spearman correlation coefficient *r* = −0.3588 (IC95 −0.4962; −0.039, *p* < 0.0001). There was a significant difference in Delta_DLCO_ between men (median difference = 10) and women (median difference = 4, *p* < 0.0001).

Although more patients were diagnosed as having altered *D*_LCO_ with GLI, the severity of alteration was milder. Malinovschi et al. (2020) compared *D*_LCO_ (%pred) in 4,903 healthy never-smoking, middle-aged adults from the SCAPIS cohort. They found that the GLI LLN for *D*_LCO_ was lower than the estimated LLN by lambda–mu–sigma (LMS) method. Individuals with *D*_LCO_ above the GLI LLN but below the SCAPIS LLN had, to a larger extent, an increased respiratory burden. In chronic respiratory diseases, Wapenaar et al. (2019) showed that using GLI reference equations significantly enhances the number of patients who are eligible to clinical studies with a *D*_LCO_ threshold >30%. Brazzale et al. (2020) showed on a retrospective study on 33,863 *D*_LCO_ measures that, when using GLI equations, results were reclassified from abnormal to normal more frequently for younger adults and for female adults and that this effect was of different size depending on the *D*_LCO_ reference equation used (Crapo, Miller, or Roca). As for all lung function measurements (Bhatt et al., 2019), it is essential that appropriate reference values are used and that the criteria used to define altered *D*_LCO_ associate with clinical outcomes. This point is not trivial since authors do not always report the reference equations used for diagnosing abnormal *D*_LCO_ (Mo et al., 2020), and some authors use a fixed cutoff value of 80% (Mo et al., 2020), whereas others use the 5th centile or LLN (Frija-Masson et al., 2020). In addition, in our cohort, 49% of the subjects were Caucasian; thus, the prevalence obtained in other countries might not be the same.

Of note, the Global Lung Function Initiative very recently published an erratum on the reference equation for *D*_LCO_ that affected the predicted values for female adult and for the calculation of z-scores in female adults (Stanojevic et al., 2020). These modified equations are not implemented in most PFT software to date and thus cannot be used by clinicians, but the differences are minimal.

### Current Knowledge on Diffusion Capacity Alteration After COVID-19

Limited data exist on pulmonary function in SARS-CoV-2 survivors. In the study by Mo et al. (2020), at hospital discharge, anomalies were noted in *D*_LCO_ (% pred) in 51 cases (47.2%) of the 110 patients. There was a significant difference in impaired diffusing capacity among the different groups of severity, which accounted for 30.4% in mild illness, 42.4% in pneumonia, and 84.2% in severe pneumonia, respectively (*p* < 0.05). In the study by Frija-Masson et al. (2020), at 1 month after symptom onset, median *D*_LCO_ was 80 (Q1 70; Q3 92), but 30% (15/50) of the patients had altered diffusion capacity (defined as *D*_LCO_ < LLN). There was no difference in *D*_LCO_ (% pred values) between groups of CT extent but a significant difference in the proportion of abnormal values (*p* = 0.0277). Lower *D*_LCO_ (% predicted value) was significantly associated with older age (>50 years) (*p* = 0.0351); there was no significant difference between groups of clinical severity (i.e., oxygen requirement). In a study by Zhao et al. (2020a), among 55 patients evaluated at 3 months after symptom onset, 71% had abnormal chest CT, but only 16% of patients had *D*_LCO_ < 80% pred. Of note, in this study, only four patients had severe pneumonia (i.e., requiring oxygen), and four patients were included but had no radiological pneumonia. The higher prevalence of diffusion capacity alteration in our data is likely explained by the inclusion of 17% of patients who had required invasive ventilation. In the prospective study by Shah et al. (2020), 58% of the 60 included patients had abnormal *D*_LCO_ at 3 months, and 88% of them had abnormal chest CT.

Data are absent for patients with chronic respiratory diseases. Hu et al. (2020) reported that COPD increases all-cause mortality in patients with COVID-19, but no functional data during follow-up were available.

Altogether, these studies highlight the fact that more than half of the patients have altered *D*_LCO_ after SARS-CoV-2 infection and that lower *D*_LCO_ is related to older age and severe-to-extremely severe radiological pneumonia. Pre-SARS-CoV-2 pulmonary function was not available in published series, but most patients included were devoid of chronic respiratory diseases. In a recent meta-analysis on 378 survivors of MERS and SARS-CoV, Ahmed et al. (2020) report that *D*_LCO_ < 80% pred has a pooled estimate of 24.35 (95% confidence interval, 11.05–45.46) at 6 months. Despite a much smaller number of affected patients worldwide and the inclusion of only severe cases in the meta-analysis, this is markedly lower than in SARS-CoV-2 survivors.

## Alteration of *D*_LCO_: Vascular Disease, Fibrosis, or Both?

### Association Between Altered *D*_LCO_ and Restriction

In studies assessing pulmonary function at 1 and 3 months (Frija-Masson et al., 2020; Mo et al., 2020; Shah et al., 2020; Zhao et al., 2020a), altered *D*_LCO_ was the most common abnormality and was often accompanied by restrictive disorder. Restriction can result from reduction in chest wall compliance, reduction in lung compliance, inspiratory muscle weakness, or a combination thereof. Interestingly, any of these pathophysiological alterations may be present in survivors of severe COVID-19 due to COVID-specific extensive lung damage or myositis or complications of prolonged intensive care such as diaphragm dysfunction associated with critical illness myopathy (Petrof, 2018). In the study by Mo et al. (2020), 25% of patients had TLC < 80%, but the authors do not use LLN to diagnose restriction and do not report specifically patients with altered *D*_LCO_ and TLC; nonetheless, mean kCO (*D*_LCO_/VA) was normal (92% pred value), and TLC and kCO were significantly lower in patients with severe pneumonia. In the study by Zhao et al. (2020a), altered *D*_LCO_ alone at 3 months was the most frequent pathological finding (16.36% of patients), but restriction and diffusion alteration were present in only 5.45% of patients. There was a significant correlation with initial D-dimer level, which suggests that vascular thrombosis and/or embolism may contribute to *D*_LCO_ reduction (Zhao et al., 2020a). In the data we present here, at 3 months, there was a significant difference in TLC (*p* < 0.0001) and *D*_LCOGLI_ (*p* < 0.0001) but not kCO for patients with residual ground glass opacities compared with normal CT at 3 months. There was a significant difference in TLC between patients with and without obesity (*p* = 0.0167) but not in *D*_LCOGLI_. Radiological emphysema was present in a minority of patients and is unlikely to account for a significant proportion of altered *D*_LCO_. This result suggests that alveolar lesions are a key determinant of reduced lung function. Thus, it is unclear if altered *D*_LCO_ up to 3 months after COVID-19 pneumonia reflects persistent alteration of the alveolar–capillary membrane, reduced lung volumes, or other mechanisms in COVID-19 survivors.

### Autopsy Series and Case Reports

In the radiological case series by Zhao et al. (2020b), the most frequent feature during acute phase was ground glass opacities (GGO), either isolated GGO (86.1%) or mixed GGO and consolidation (64.4%). This was followed by vascular enlargement in the lesion (71.3%) and traction bronchiectasis (52.5%). At discharge, Wang et al. (2020) show that most patients present with consolidation of lesions, with fibrotic lesions remaining only in 12% of cases. Critically ill patients had more often consolidation and bilateral lung involvement (Qian et al., 2020). Unfortunately, most case series reporting radiological fibrosis after COVID-19 included a small number of patients, most who had required mechanical ventilation (either invasive or non-invasive), which in itself can cause lung injury (Fang et al., 2020; Huang et al., 2020).

Combet et al. (2020) report the case of a 38-year-old man who presented with extensive pulmonary honeycombing fibrosis in territories where GGO had been initially present, 10 days after symptom onset, and without invasive ventilation. Similarly, Schwensen et al. (2020) report the case of a 80 years old woman who had normal chest CT prior to infection and died of diffuse lung fibrosis.

Several histopathological series have been published (Magro et al., 2020; Menter et al., 2020). Although all reported a high prevalence of microthrombi and vascular lesions in deceased patients, the presence of alveolar damage was inconsistent. Polak et al. reviewed 129 cases of published lung samples (either full/partial autopsy or lung resection) and identified three main histological patterns: epithelial (*n* = 110, 85%), with reactive epithelial changes and diffuse alveolar damage (DAD); vascular (*n* = 76, 59%) with microvascular damage, (micro)thrombi, and acute fibrinous and organizing pneumonia; and fibrotic (*n* = 28, 22%) with interstitial fibrosis. The epithelial and vascular patterns were present in all stages, whereas the fibrotic pattern started at 3 weeks of evolution. Patients could present with more than one pattern, either simultaneously or consecutively. Unfortunately, chest CT results were not reported; the presence of fibrosis was not associated with mechanical ventilation.

These differences could be explained by different inclusion criteria (deceased patients vs. lung sample), number of cases in the series, and difference in time from diagnosis to lung specimen. Indeed, most patients died after several days or weeks under ventilator support, which can lead to lung injury despite protective measures (Slutsky and Ranieri, 2013).

### Vasculopathy, Fibrosis, or Both?

The key receptor to SARS-CoV-2 entry, angiotensin−converting enzyme (ACE)-2, is expressed on pneumocytes and macrophages, as well as on the surface of arterial endothelial and smooth muscle cells of the lungs (Hamming et al., 2004). Endothelial dysfunction induced by SARS-CoV-2 creates a favorable environment for thrombosis (Evans et al., 2020), which in turn can favor inflammation, representing the immunothrombosis model (Gaertner and Massberg, 2016). ACE-2 has been shown to be activated in acute lung injury and linked to acute respiratory distress syndrome (ARDS) severity (Orfanos et al., 2000; Jerng et al., 2006). Using a combined *in vitro* and *in silico* approach, Xu et al. (2020) showed that SARS-CoV-2 induces transcriptional signatures in human lung epithelial cells that promote lung fibrosis, such as TMPRSS2, ADAM metallopeptidase domain 17 (ADAM17), tissue inhibitor of metalloproteinase 3 (TIMP3), angiotensinogen, transforming growth factor beta 1 (TGFB1), connective tissue growth factor (CTGF), vascular endothelial growth factor A (VEGF A), and fibronectin.

Thus, is it possible to explain alteration of *D*_LCO_ by both fibrosis and vascular disease?

In infiltrative lung diseases (ILDs), Probst et al. (2020) recently reviewed the evidence of vascular involvement in fibrosis progression. In systemic sclerosis and idiopathic pulmonary fibrosis (IPF), there is an increase in vascular permeability even during early stages of the disease. There is also evidence for a vascular remodeling and a key role for the hematopoietic-vascular niche in fibrosis promotion (Cao et al., 2016).

As exerted by Eapen et al. (2020), endothelial-to-mesenchymal transition (EndMT) can occur when endothelial cells respond to injury and transform themselves in a more aggressive mesenchymal state. The authors point out that histopathological findings in fatal COVID-19 cases reveal important vascular changes that are compatible with EndMT and that this is consistent with the different receptors that facilitate SARS-CoV-2 entry in endothelial cells. The disruption of endothelial cells induced by EndMT facilitates cell migration and can trigger fibrosis.

Neutrophils extracellular traps (NETs) are DNA fibers decorated with proteins normally confined to granules, including antimicrobial molecules. They are implicated in lung damage by promoting differentiation and function of fibroblasts (*in vitro*) (Chrysanthopoulou et al., 2014), thrombosis, and can be formed in response to numerous infectious and non-infectious stimuli (Boeltz et al., 2019). The presence of NET has been confirmed in the lungs of patients with severe COVID-19, infiltrating airways, interstitium, and vascular compartment (Radermecker et al., 2020). This ubiquitous presence could result in vascular damage and fibrosis altogether, but all four patients had been under mechanical ventilation and died of respiratory failure, and these findings might not be generalized to all COVID-19 patients. Bendib et al. (2019) found NETs in bronchoalveolar lavage and blood of patients with ARDS but no significant relationship between bronchoalveolar lavage neutrophil extracellular trap concentrations and ventilator-free days.

## Conclusion and Perspectives

Alteration of diffusion capacity of the lung is frequent after SARS-CoV-2 infection, although its prevalence and severity depend on the reference equation used. The use of GLI reference equations should be strongly encouraged. It is still unclear if this alteration results from vascular disease (including thrombopathy), fibrotic sequelae, respiratory muscle weakness, or a combination of these factors.

This high prevalence of altered *D*_LCO_ will induce a high number of patients to follow after infection has resolved and put a pressure on pulmonologists. Giving a wide access to PFT will be crucial (Andrejak et al., 2020; Raghu and Wilson, 2020; Rovere Querini et al., 2020), but better knowledge on the natural history of COVID-19 could help selecting the patients who may benefit from close and repeated follow-up of *D*_LCO_. Different countries might choose different follow-up algorithms, depending not only on their capacity to give access to full PFT (including FRC and *D*_LCO_) or spirometry only, but also on the ongoing epidemics that will affect access to PFT and PFT procedures (Ers Group 9.1 and Ers Group 4.1, 2020; Rovere Querini et al., 2020; Wilson et al., 2020). To prevent overwhelming of PFT laboratories, chest CT and/or X-ray have been proposed as screening tools (George et al., 2020), but X-ray might not have a high sensitivity to detect sequelae. In addition, these algorithms often rely on persistent symptoms to decide further investigations, and it has been established that dyspnea is present in at least 50% of patients at 3 months, including those with mild initial symptoms (Garrigues et al., 2020; Goërtz et al., 2020). Tools assessing functional status and quality of life are of great importance when evaluating the cost of COVID-19 on patients’ life (Klok et al., 2020). A common frame of surveillance and outcome measures is needed to have access to comparable data worldwide (Patel et al., 2020). The effect of COVID-19 on patients with chronic respiratory diseases needs to be assessed in multicentric cohorts. Finally, the effect of different treatments, particularly steroids, needs to be assessed.

## Data Availability Statement

The raw data supporting the conclusions of this article will be made available by the authors, without undue reservation, in respect of the GDPR.

## Ethics Statement

The studies involving human participants were reviewed and approved by the Institutional Review Board of the French learned society for respiratory medicine – Société de Pneumologie de Langue Française. The patients/participants provided their non opposition to participate in this study.

## Author Contributions

JF-M: acquisition of data, manuscript writing, and statistics. CB, HB, LM, DP, and MF: acquisition of data, manuscript correction, and final approval. LP and M-Pd’O: manuscript writing and final approval. All authors contributed to the article and approved the submitted version.

## Conflict of Interest

The authors declare that the research was conducted in the absence of any commercial or financial relationships that could be construed as a potential conflict of interest.
